# Association of opioid use disorder with outcomes of hospitalizations for acute myocardial infarction in the United States

**DOI:** 10.1016/j.clinsp.2023.100251

**Published:** 2023-07-18

**Authors:** Nameer Ascandar, Amulya Vadlakonda, Arjun Verma, Nikhil Chervu, Jacob S. Roberts, Sara Sakowitz, Catherine Williamson, Peyman Benharash

**Affiliations:** Cardiovascular Outcomes Research Laboratories, Department of Surgery, David Geffen School of Medicine at UCLA, Los Angeles, California, USA

**Keywords:** Opioid use disorder, Acute myocardial infarction, Cardiovascular, Readmissions, Percutaneous coronary intervention, Coronary artery bypass graft

## Abstract

**Objective:**

While Opioid Use Disorder (OUD) has been linked to inferior clinical outcomes, studies examining the clinical outcomes and readmission of OUD patients experiencing Acute Myocardial Infarction (AMI) remain lacking. The authors analyze the clinical and financial outcomes of OUD in a contemporary cohort of AMI hospitalizations.

**Methods:**

All non-elective adult (≥ 18 years) hospitalizations for AMI were tabulated from the 2016‒2019 Nationwide Readmissions Database using relevant International Classification of Disease codes. Patients were grouped into OUD and non-OUD cohorts. Bivariate and regression analyses were performed to identify the independent association of OUD with outcomes after non-elective admission for AMI, as well as subsequent readmission.

**Results:**

Of an estimated 3,318,257 hospitalizations for AMI meeting study criteria, 36,057 (1.1%) had a concomitant diagnosis of OUD. While OUD was not significantly associated with mortality, OUD patients experienced superior cardiovascular outcomes compared to non-OUD. However, OUD was linked to increased odds of non-cardiovascular complications, length of stay, costs, non-home discharge, and 30-day non-elective readmission.

**Conclusions:**

Patients with OUD presented with AMI at a significantly younger age than non-OUD. While OUD appears to have a cardioprotective effect, it is associated with several markers of increased resource use, including readmission. The present findings underscore the need for a multifaceted approach to increasing social services and treatment for OUD at index hospitalization.

## Highlights

• Patients with opioid use disorder experience greater in-hospital mortality and readmissions following MI.
• Paradoxically, these patients experience superior cardiovascular outcomes compared to their counterparts.
• Opioid use disorder remains associated with high resource utilization and healthcare expenditures.

## Introduction

The misuse of opioids in the United States is a growing crisis with nearly 47,600 attributable deaths in 2017.^1^ The widespread prevalence of Opioid Use Disorder (OUD) has previously been linked to several structural determinants, including socioeconomic status, race, economic opportunity, and social cohesion.^2^ A recent retrospective study found that cardiovascular disease is the second most common cause of mortality in patients with OUD, second only to direct drug poisoning.^3^ Yet, associations between cardiovascular outcomes and OUD are under-examined.

The effects of chronic opiate exposure on the cardiovascular system remain controversial. Several *in vivo* and *in vitro* studies have demonstrated the cardioprotective and anesthetic preconditioning effects of opioids, mediated primarily by delta receptor activation and protein kinase C transduction pathways.^4^, ^5^, ^6^ In fact, Marmor reported OUD to be associated with a lower risk of significant Coronary Artery Disease (CAD) ^7^. Conversely, several recent clinical and population studies have challenged this association, showing a significantly increased risk of Acute Myocardial Infarction (AMI) and arrhythmia in patients with prolonged opioid use, seen in as few as 6 months.^6^^,^^8^, ^9^, ^10^ In a study of 2253 propensity-matched pairs, Ranka and coworkers noted OUD to be associated with increased complications but not mortality after an AMI.^11^ However, this study suffers from several limitations, including a lack of information on associated hospitalization costs and readmissions.

In the present work, the authors examined the clinical and financial outcomes of OUD in a national cohort of AMI hospitalizations. The authors hypothesized OUD to be independently associated with a greater risk of complications but not in-hospital mortality. The authors further hypothesized increased readmissions within 30 days of index discharge and hospitalization costs among the OUD cohort.

## Methods

All adult (≥ 18 years) non-elective admissions for AMI, classified as ST-Segment Elevation (STEMI) or non-ST-Segment Elevation Myocardial Infarction (NSTEMI) were abstracted from the 2016‒2019 Nationwide Readmissions Database (NRD). The NRD is the largest all-payer readmissions database in the US and provides accurate estimates for ∼60% of all hospitalizations.^12^ The NRD is maintained by the Agency for Healthcare Research and Quality's Healthcare Cost and Utilization Project. Unique patient identifiers allow for tracking of re-hospitalizations within the state over each calendar year. *International Classification of Disease, Tenth Revision* (ICD-10) codes (Supplemental Table 1) were used to identify relevant diagnoses while additional patient and hospital characteristics were defined according to the NRD data dictionary. This included age, sex, income quartile, primary insurance, hospital teaching status, and bed size. Records missing data for age, in-hospital mortality, and costs were excluded (0.46%). Those with a primary diagnosis of infective endocarditis or type 2 AMI were similarly excluded (14.3%).

The primary outcome of interest was in-hospital mortality and cardiac complications, which included cardiogenic shock, ventricular tachycardia/fibrillation, cardiac arrest, acute heart failure, and other cardiac complications. Other cardiac complications were considered as a composite of cardiac tamponade, pericarditis, hemopericardium, atrial and ventricular septal defect, chordae tendinea rupture, papillary muscle rupture, angina, intracardiac thrombus, and ventricular wall rupture. Secondary outcomes included respiratory, infectious, thromboembolic, acute kidney injury, and neurologic complications, and key markers of resource use. Specifically, the authors considered Length Of Stay (LOS), hospitalization costs, non-home discharge (defined as discharge to a skilled nursing facility, acute care facility, intermediate care facility, or against medical advice), and 30-day non-elective readmissions. Hospitalization costs were converted from charges using center-specific cost-to-charge ratios and adjusted for inflation to the 2019 Personal Health Index. Diagnosis-related groups were used to identify admitting diagnoses upon re-hospitalization.

Categorical variables are reported as proportions while normally distributed continuous variables are shown as means with standard deviation. Non-normally distributed continuous variables are reported as median and interquartile range. The significance of intergroup differences of continuous variables was assessed using the adjusted Wald and Mann Whitney *U* tests, as appropriate. Pearson's Chi-Square test was used to compare categorical variables. The authors used Cuzick's non-parametric test to assess the significance of temporal trends in the study (NPtrend).^13^ Prior to regression, entropy balancing was used to obtain a weighted comparison group with comparable covariate distributions. Entropy balancing has several advantages over traditional propensity score matching and preserves the entire cohort for further analysis.^14^

Model covariates were selected based on clinical relevance. Subsequent multivariable mixed regressions were developed to examine the association of OUD with described outcomes of interest. Models were adjusted for age, sex, and concomitant substance use disorder, among other factors (Supplemental Table 2). Models were Optimized Using Receiver-Operating Characteristics (AUROC) as well as Akaike and Bayesian Information Criteria to reduce overfitting. Regression outputs are reported as Adjusted Odds Ratios (AOR) or Beta (β) coefficients, both with 95% Confidence Intervals (95% CI). An α less than 0.05 was considered significant. Stata 16.1 (StataCorp, College Station, TX) was used for all statistical analyses. Given the deidentified nature of the data, this study was deemed exempt from full review by the Institutional Review Board at the University of California, Los Angeles. The Strengthening the Reporting of Observational Studies in Epidemiology (STROBE) reporting guideline was followed for this study.

## Results

Of an estimated 3,318,257 hospitalizations for AMI included for analysis, 36,057 (1.1%) had a concomitant diagnosis of OUD. The prevalence of OUD modestly decreased from 11.0 per 1,000 hospitalizations in 2016 to 10.5 per 1,000 hospitalizations in 2019 (NPtrend < 0.01).

Compared to others, the OUD cohort was younger (59 [50‒67] vs. 69 [59‒79] years, *p* < 0.001), and more commonly female (41.9% vs. 39.9%, *p* < 0.001). Specifically, OUD patients more commonly suffered from chronic lung disease (37.2% vs. 23.9%, *p* < 0.001), liver disease (15.1 vs. 5.1%, *p* < 0.001), and psychiatric disorders not related to substance use (24.2% vs. 10.5%, *p* < 0.001). Furthermore, concomitant substance uses disorders, including tobacco (62.2% vs. 46.4%, *p* < 0.001), alcohol (12.4% vs. 3.8%, *p* < 0.001), and non-opioid drug use (28.1% vs. 3.2%, *p* < 0.001) were more prevalent in the OUD cohort compared to the non-OUD group. Compared to others, OUD had higher rates of NSTEMI (82.8% vs. 75.8%, *p* < 0.001). OUD patients were more frequently treated at a metropolitan teaching hospital (72.1% vs. 69.7%, *p* < 0.001), and were more often covered by Medicaid (27.3% vs. 8.4%, *p* < 0.001), compared to their counterparts (Table 1).

**Table 1:** Demographic, clinical, and hospital characteristics.

	Non-OUD (n= 3,282,200)	OUD (n=36,057)	p-value
**Age (years [IQR])**	69 [59‒79]	59 [50‒67]	<0.001
**Female (%)**	39.9	41.9	<0.001
**Type of MI (%)**			<0.001
STEMI	24.2	17.2	
NSTEMI	75.8	82.8	
**Income quartile (percentile, %)**			<0.001
76‒100^th^	16.7	12.5	
51‒75^th^	23.8	22.0	
26‒50^th^	28.6	28.3	
0‒25^th^	30.9	37.3	
**Insurance Status (%)**			<0.001
Private	21.8	14.3	
Medicare	62.7	48.9	
Medicaid	8.4	27.3	
Other Payer	7.1	9.5	
**Hospital teaching status (%)**			<0.001
Non-Metropolitan	7.7	5.3	
Metropolitan non-teaching	22.6	22.6	
Metropolitan teaching	69.7	72.1	
**Hospital bed size (%)**			0.410
Small	14.8	14.3	
Medium	28.1	29.0	
Large	57.0	56.7	
**Comorbidities (%)**			
Congestive heart failure	29.9	29.0	0.016
Valve disease	15.4	10.9	<0.001
Coagulopathy	8.4	11.3	<0.001
Chronic lung disease	23.9	37.2	<0.001
Pulmonary hypertension	6.7	6.9	0.29
Peripheral vascular disease	12.8	10.7	<0.001
Hypertension	79.7	69.0	<0.001
Diabetes	39.8	30.6	<0.001
Hypothyroidism	12.7	9.7	<0.001
Anemia	4.4	5.2	<0.001
Electrolyte abnormality	33.8	48.9	<0.001
End-stage renal disease	5.9	4.8	<0.001
Liver disease	5.1	15.1	<0.001
Weight loss	6.0	10.2	<0.001
Neurological disorders	12.5	23.0	<0.001
Psychiatric disorder, excluding SUD	10.5	24.2	<0.001
**Comorbid Substance Use Disorder (%)**			
Alcohol Use Disorder	3.8	12.4	<0.001
Non-opioid drug use disorder	3.2	28.1	<0.001
Tobacco use disorder	46.4	62.2	<0.001

IQR, Interquartile Range; NSTEMI, Non-ST-Elevation Myocardial Infarction; SD, Standard Deviation; STEMI, ST-Elevation Myocardial Infarction; SUD, Substance Use Disorder

Data are reported as proportions unless otherwise noted. Statistical significance was set at α = 0.05.

Other Payer includes self-pay, no charge, and other payer, as defined by the NRD.

On bivariate analysis, patients with OUD demonstrated similar rates of in-hospital mortality (8.6% vs. 8.6%, *p* = 0.79) compared to others. Although the OUD cohort experienced higher rates of cardiac arrest (5.9% vs. 4.1%, *p* < 0.001), they had lower rates of cardiogenic shock (6.0% vs. 6.4%, *p* = 0.030), ventricular tachycardia/fibrillation (8.6% vs. 9.2%, *p* = 0.017), and acute heart failure (23.8% vs. 24.8%, *p* = 0.008). There was no difference in rates of other cardiac complications (2.0% vs. 2.1%, *p* = 0.21), compared to others. The OUD cohort less frequently required revascularization procedures with percutaneous coronary intervention (PCI, 17.8% vs. 29.7%, *p* < 0.001) or coronary artery bypass graft (CABG, 5.5% vs. 7.7%, *p* < 0.001), compared to non-OUD. A higher proportion of OUD patients sustained acute kidney injury (35.1% vs. 25.8%, *p* < 0.001) among other complications (Table 2). The OUD cohort had higher resource utilization than the non-OUD cohort, with increased LOS (5 [3‒9] vs. 3 [2‒7] days, *p* < 0.001) and higher hospitalization costs ($18,600 [$11,000‒$33,100] vs. $18,100 [$10,700‒$30,600], *p* < 0.001). Moreover, OUD had higher rates of non-elective 30-day readmission (16.1% vs. 11.3%, *p* < 0.001) and more frequently left against medical advice (5.4% vs. 1.1%, *p* < 0.001).

**Table 2:** Unadjusted outcomes following index hospitalization stratified by OUD.

	Non-OUD (n=3,282,200)	OUD (n=36,057)	p-value
**Mortality (%)**	8.6	8.6	0.79
**Major Complications (%)**			
Cardiogenic shock	6.4	6.0	0.030
Ventricular Tachycardia/Fibrillation	9.2	8.6	0.017
Cardiac Arrest	4.1	5.9	<0.001
Acute Heart Failure	24.8	23.8	0.008
Other Cardiac Complications	2.1	2.0	0.21
Infectious	10.9	19.3	<0.001
Respiratory	15.0	21.7	<0.001
Thromboembolic	1.4	2.0	<0.001
Acute kidney injury	25.8	35.1	<0.001
Stroke	0.8	0.9	0.14
**Non-elective 30-day readmission (%)**	11.3	16.1	<0.001
**Discharge disposition (%)**			<0.001
Home	57.5	53.1	
Short-term facility	2.8	2.6	
Long-term facility	15.5	15.8	
Home healthcare	14.5	14.5	
Against medical advice	1.1	5.4	
Other^a^	8.6	8.6	
**Resource Utilization**			
Length of stay (days) [IQR]	3 [2‒7]	5 [3‒9]	<0.001
Cost (USD $1,000) [IQR]	18.1 [10.7‒30.6]	18.6 [11.0‒33.1]	<0.001
**Revascularization Procedures (%)**			
Percutaneous Coronary Intervention	29.7	17.8	<0.001
CABG	7.7	5.5	<0.001

IQR, Interquartile Range; OUD, Opioid Use Disorder; USD, United States dollar; CABG, Coronary Artery Bypass Graft.

Data are presented as percentage or median [IQR], unless otherwise indicated.

Other cardiac complications include Cardiac Tamponade, Pericarditis, Hemopericardium, Atrial Septal Defect, Ventricular Septal Defect, Chordae Tendinea Rupture, Papillary Muscle Rupture, Angina, Intracardiac Thrombus, Ventricular Wall Rupture.

a Comprises in-hospital mortality, transfer to law enforcement, and discharge to an unknown destination.

**Fig. 1:**
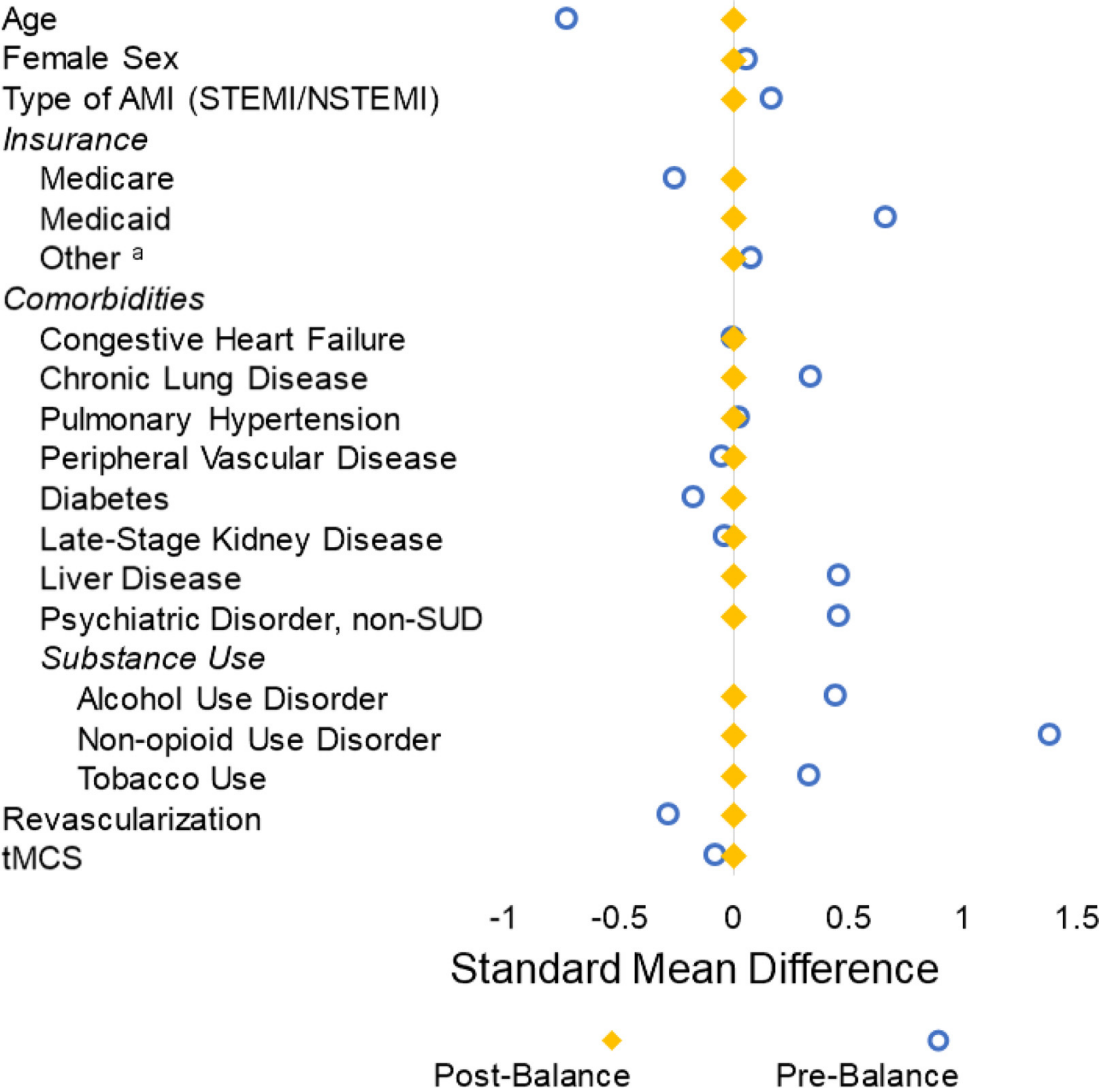
Forrest plot demonstrates adequate covariate balancing between groups. AMI, Acute Myocardial Infarction; STEMI, ST-Elevation Myocardial Infarction; NSTEMI, Non-ST-Elevation Myocardial Infarction; non-SUD, Non-Substance Use Disorder; tMCS, Temporary Mechanical Circulatory Support; NRD, Nationwide Readmissions Database. *Other Payer includes self-pay, no charge, and other payer, as defined by the NRD.

Entropy balancing resulted in adequate covariate balance, as shown in Fig. 1. On risk-adjusted analysis, OUD did not alter the odds of in-hospital mortality (AOR = 1.06, 95% CI 0.99‒1.13). The authors also identified significant differences in cardiovascular outcomes between the OUD and non-OUD cohorts. Namely, while OUD patients had higher adjusted odds of cardiac arrest (AOR = 1.21, 95% CI 1.13‒1.29), they had lower odds of other cardiac complications (AOR = 0.88, 95% CI 0.79‒0.98). There was no difference in cardiogenic shock (AOR = 1.05, 95% CI 0.98‒1.13) ventricular tachycardia/fibrillation (AOR = 0.98, 95% CI 0.93‒1.04), or acute heart failure (AOR = 0.99, 95% CI 0.95‒1.04). between cohorts. In regard to non-cardiovascular outcomes, the OUD cohort had higher adjusted odds of acute kidney injury (AOR = 1.50, 95% CI 1.44‒1.55), infectious complications (AOR = 1.70, 95% CI 1.63‒1.77), and respiratory complications (AOR = 1.42, 95% CI 1.37‒1.48).

However, OUD remained associated with key markers of resource utilization at index hospitalization (Table 3). On adjusted analysis with non-OUD as a reference, OUD demonstrated longer hospitalizations (*β* = 1.41, 95% CI 1.25‒1.56) and a $2,800 (95% CI $2,200‒$3,400) increment in healthcare expenditures. Furthermore, OUD was linked to increased odds of non-home discharge (AOR = 1.38, 95% CI 1.33‒1.43) and non-elective 30-day readmission (AOR = 1.34, 95% CI 1.28‒1.40).

**Table 3:** Adjusted outcomes following AMI for OUD compared to non-OUD, using weights derived from entropy balancing.

	OUD	95% CI	p-value
**Clinical outcomes (AOR)**			
In-hospital mortality, index hospitalization	1.06	0.99 – 1.13	0.06
Cardiogenic shock	1.05	0.98 – 1.13	0.16
Ventricular Tachycardia/Fibrillation	0.98	0.93 – 1.04	0.57
Cardiac Arrest	1.21	1.13 – 1.29	<0.001
Acute Heart Failure	0.99	0.95 – 1.04	0.70
Other Cardiac Complications	0.88	0.79 – 0.98	0.020
Stroke	1.12	0.96 – 1.30	0.16
Thromboembolic Complications	1.13	1.01 – 1.27	0.033
Infectious Complications	1.70	1.63 – 1.77	<0.001
Respiratory Complications	1.42	1.37 – 1.48	<0.001
Acute Kidney Injury	1.50	1.44 – 1.55	<0.001
**Resource Utilization (β)**			
Length of stay (days)	+1.41	1.25 – 1.56	<0.001
Cost (USD $1,000)	+2.83	2.26 – 3.40	<0.001
Non-Home discharge (AOR)	1.38	1.33 – 1.43	<0.001
Non-elective 30-day readmission (AOR)	1.34	1.28 – 1.40	<0.001

At the first non-elective readmission, OUD patients demonstrated a lower unadjusted rate of in-hospital mortality (3.6% vs. 6.4%, *p* < 0.001), as shown in Table 4. The OUD cohort was less commonly readmitted for cardiovascular-related conditions (32.1% vs. 40.4%, *p* < 0.001). Patients with OUD had lower rates of stent placement (5.0% vs. 7.0%, *p* < 0.001) but no difference in CABG (0.8% vs. 1.1%, *p* = 0.14) or valve repair/replacement (0.5% vs. 0.4%, *p* = 0.58) during re-hospitalization. At readmission, there was no difference in duration of hospitalization (4 [2‒7] vs. 4 [2‒7] days, *p* = 0.31) or hospitalization costs ($9,500 [$5,500‒$17,700] vs. $9,500 [$5,400‒$18,500], *p* = 0.93).

**Table 4:** Unadjusted outcomes at first non-elective readmission within 30-days of index discharge.

	Non-OUD	OUD	p-value
	(n=3,282,200)	(n=36,057)	
**In-hospital mortality (%)**	6.4	3.6	<0.001
**Reason for readmission (%)**			
Cardiovascular	40.4	32.1	<0.001
CABG	1.1	0.8	0.14
Stent Placement	7.0	5.0	<0.001
Valve repair/replacement	0.4	0.5	0.58
Acute kidney injury	3.3	3.6	0.27
Respiratory	9.5	12.2	<0.001
Endocrine	2.8	3.4	0.07
Gastrointestinal	7.8	6.3	0.003
Infection	11.5	12.4	0.14
Hematological	1.4	1.1	0.18
Skin/soft tissue	1.4	5.0	<0.001
Neurological	3.3	2.9	0.26
Psychiatric disorder	0.7	2.5	<0.001
**Length of stay (days) [IQR]**	4 [2‒7]	4 [2‒7]	0.31
**Cost (USD $1,000) [IQR]**	9.5 [5.4‒18.5]	9.5 [5.5‒17.7]	0.93

OUD, Opioid Use Disorder; USD, United States Dollar.

Outcomes reported as proportions.

Cardiovascular: includes all cardiovascular related diagnoses.

## Discussion

In the context of the nearly five-fold increase in US medical hospitalizations for OUD over the past two decades,^15^ the present study examined outcomes of 36,057 patients with OUD admitted with AMI. Although OUD patients were younger, they had a higher burden of several chronic medical conditions, but no difference in mortality compared to others. However, patients with OUD experienced lower rates of cardiovascular complications and procedural intervention at index hospitalization. Nonetheless, OUD appears associated with several indicators of increased healthcare expenditures, including in-patient costs, LOS, non-home discharge, and 30-day readmissions. Several of these findings warrant further discussion.

As expected, given their younger age at presentation, patients with OUD less frequently had age-related diagnoses of hypertension, diabetes, and peripheral vascular disease. Conversely, they had a higher burden of chronic lung and liver diseases. Such comorbidities may be sequelae of chronic polysubstance use,^16^ which was present in nearly 75% of OUD patients analyzed. Although the authors were unable to examine the extent of coronary artery disease in the present study, OUD patients less frequently presented with STEMI and required fewer revascularization procedures compared to non-OUD patients, suggesting a less severe AMI course. The relative absence of other major age-related comorbidities may explain the reduced cardiovascular complications among OUD patients.

Animal studies have generated hypotheses that could explain why opioids may be paradoxically cardioprotective. Opiates have been implicated in Ischemic Preconditioning (IPC) in animal models. IPC is a process by which brief periods of coronary artery occlusion and reperfusion activate opioid receptors δ and μ and stimulate signal transduction via protein kinase C.^17^ This has been found to reduce the size of infarcted myocardial tissue and mitigate the resultant ischemic injury in isolated rat hearts.^18^, ^19^, ^20^ Since these effects have been primarily described in animal models, the true effects of long-term opioid exposure on the conditioning of the cardiovascular system remain to be elucidated in OUD patients. Nonetheless, acute opioid administration (intravenous morphine sulfate) has been the recommended treatment for cardiac pain refractory to nitroglycerine.^21^ Further studies are needed to clarify the mechanism behind the apparent cardioprotective effects of opioids.

Those with OUD presented with AMI at a much younger age, potentially suggesting a greater burden of atherosclerosis. While the younger presentation of AMI in OUD patients has not been widely studied, several investigators have noted the extent of opioid use to correlate directly with the degree of coronary artery disease, hyperlipidemia,^22^^,^^23^ and AMI rates.^8^^,^^24^ OUD has been linked to poor nutritional status, caused by both metabolic and lifestyle derangements.^25^ Specifically, the xerostomia and appetite suppression caused by opioids predisposes those with OUD to have diets high in fat and sugar.^25^^,^^26^ Furthermore, Strike et. al. found that over 50% of intravenous drug users experience food insecurity, defined as the systematic and chronic lack of access to nutritional food.^27^ Although not mechanistic, these findings may, in part, explain the accelerated presentation of AMI among opioid users.

As noted above, OUD patients had higher odds of respiratory, renal, and infectious complications. This may contribute to the longer and more expensive hospitalizations the authors found to be associated with OUD. In a study of 56,278 patients with OUD, Clark and colleagues found that the degree of comorbidities experienced by OUD patients raises the cost of their hospitalizations.^28^ As the annual opioid-related healthcare expenditures in the US reach an estimated $89.1 billion, mitigating costs and resource use for OUD patients is increasingly relevant.^29^ Furthermore, OUD patients were more likely to be readmitted within 30 days, which is consistent across various elective and emergent hospitalizations.^30^, ^31^, ^32^ Similar to prior literature on AMI,^33^ cardiovascular reasons comprised a majority of all readmissions across the study population. However, OUD patients were less likely than their counterparts to have a cardiac-related readmission diagnosis. The authors noted OUD patients to be more frequently readmitted for respiratory and psychiatric diagnoses. Known complications of prolonged opioid use include opioid-induced respiratory depression and comorbid psychiatric disorders, as previously reported.^16^, ^17^, ^18^, ^19^, ^20^, ^21^, ^22^, ^23^, ^24^, ^25^, ^26^, ^27^, ^28^, ^29^, ^30^, ^31^, ^32^, ^33^, ^34^ Consequently, the present findings may indicate continued post-discharge opioid use, possibly resulting from suboptimal addiction counseling at the time of index hospitalization. A recent randomized controlled trial found that patient navigation services increased the OUD patients’ likelihood of adhering to medical treatment and finding resources to meet their social needs.^35^ Schoenfield and colleagues outlined barriers to accessing OUD treatment, which includes physician inexperience, stigma of substance use, and a lack of community resources to locally implement best practices.^36^ Multifaceted approaches to increase social services and treat OUD at index hospitalization could minimize these preventable readmissions.

The present study has several limitations inherent to its retrospective nature. The accuracy of the data is subject to potential coding errors of the NRD. As an administrative database, the NRD lacks detailed data on race, geographic region, and other demographics that are important predictive factors of OUD and associated outcomes. Furthermore, the NRD does not track patients who are readmitted in a state different from that of the index hospitalization. Therefore, it is likely that the burden of OUD among AMI hospitalizations and associated readmissions have been under-reported. Although the authors were able to adjust for various patient and hospital characteristics using validated diagnostic coding, the authors were unable to adjust for important factors that may have influenced key outcomes, due to the limited nature of the NRD. In particular, this study lacks patient-level data on medications, compliance, and extent of opioid use.

## Conclusion

In conclusion, OUD patients admitted for AMI presented with lower severity and had fewer procedural interventions with no change in mortality. However, they continue to have increased LOS, hospitalization costs, non-home discharge, and 30-day non-elective readmission. This may be the result of increased non-age-related comorbid conditions, persistent drug use, and socioeconomic disparities in spite of the potential protective effect of younger age. The present findings underscore the need for improved access to OUD treatment, as well as directed strategies to address financial and healthcare inequities.

## Author contributions

Nameer Ascandar and Amulya Vadlakonda: Conceptualization, Methodology, Data analysis, Writing - Original Draft, Generating Figures/Tables, Reviewing, Editing. Arjun Verma: Validation, Methodology, Reviewing, Editing. Nikhil Chervu: Reviewing, Editing. Jacob S Roberts: Writing. Sara Sakowitz: Reviewing, Editing. Catherine Williamson: Reviewing, Editing. Peyman Benharash: Conceptualization, Methodology, Supervision, Reviewing, Editing.

## Funding

The authors have no financial disclosures or conflicts of interest to report.

## Conflicts of interest

The authors declare no conflicts of interest.

## Appendix Supplementary materials

Image, application 1
