# Supplementary material for: Association of opioid use disorder with outcomes of hospitalizations for acute myocardial infarction in the United States
Source: Clinics (Sao Paulo). 2023 Jul 18;78:100251. doi: 10.1016/j.clinsp.2023.100251 (PMC10372160; doi:10.1016/j.clinsp.2023.100251)
Supplement: Supplementary file 1 [file mmc1.docx]

**CLINICS-D-22-00560_Supplementary Material**

**Supplemental Table 1** ICD 10 codes.

| **Diagnosis** | **ICD-10 Diagnosis Code** |
| --- | --- |
| Acute Myocardial Infarction | I21, I210, I211, I212, I213, I214, I219 |
|  | I21A, I2101, I2102, I2109, I2111 |
|  | I2119, I2121, I2129, I21A1, I21A9 |
| Opioid Use Disorder | F11 |

**Supplemental Table 2** Multivariable logistic regression model to predict in-hospital mortality following entropy balancing.

|  | **AOR** | **95% CI** | **p-value** |
| --- | --- | --- | --- |
| **Opioid Use Disorder** | 1.06 | 0.99 – 1.13 | 0.06 |
| **Age, years** | 1.01 | 1.01 – 1.01 | <0.001 |
| **Female** | 0.97 | 0.92 – 1.04 | 0.41 |
| **Type of AMI** |  |  |  |
| STEMI | Ref |  |  |
| NSTEMI | 0.39 | 0.36 – 0.42 | <0.001 |
| **Insurance** |  |  |  |
| Private | Ref |  |  |
| Medicare | 1.35 | 1.21 – 1.50 | <0.001 |
| Medicaid | 1.34 | 1.19 – 1.50 | <0.001 |
| Self-pay | 1.63 | 1.40 – 1.89 | <0.001 |
| **Comorbidities** |  |  |  |
| Congestive heart failure | 0.87 | 0.82 – 0.93 | <0.001 |
| Chronic lung disease | 1.03 | 0.97 – 1.10 | 0.34 |
| Pulmonary hypertension | 0.97 | 0.87 – 1.09 | 0.65 |
| Peripheral vascular disease | 1.12 | 1.03 – 1.22 | 0.009 |
| Diabetes | 0.75 | 0.70 – 0.80 | <0.001 |
| Late-stage kidney disease | 1.46 | 1.29 – 1.65 | <0.001 |
| Liver disease | 3.97 | 3.72 – 4.24 | <0.001 |
| Psychiatric disorder, excluding SUD | 0.65 | 0.60 – 0.70 | <0.001 |
| Alcohol use disorder | 0.92 | 0.83 – 1.01 | 0.08 |
| Nonopioid use disorder | 0.77 | 0.70 – 0.84 | <0.001 |
| Tobacco use | 0.50 | 0.47 – 0.54 | <0.001 |
| Revascularization with PCI or CABG | 0.22 | 0.20 – 0.24 | <0.001 |
| Temporary mechanical support | 3.38 | 2.94 – 3.88 | <0.001 |
